# Comparison of discrimination for cardio-metabolic risk by different cut-off values of the ratio of triglycerides to HDL cholesterol

**DOI:** 10.1186/s12944-019-1098-0

**Published:** 2019-07-27

**Authors:** Ichiro Wakabayashi, Takashi Daimon

**Affiliations:** 1 Department of Environmental and Preventive Medicine, Hyogo College of Medicine, Mukogawa-cho 1-1, Nishinomiya, Hyogo 663-8501 Japan; 2 Division of Biostatistics, Hyogo College of Medicine, Nishinomiya, Hyogo 663-8501 Japan

**Keywords:** Cardiovascular disease, Cut-off values, Multiple risk factors, Receiver operating characteristic, Triglycerides-to-HDL cholesterol ratio

## Abstract

**Background:**

The ratio of triglycerides to HDL cholesterol (TG/HDL-C ratio) is known as a good predictor for cardiovascular disease. The purpose of this study was to compare discrimination for cardiovascular risk by different cut-off values of the TG/HDL-C ratio.

**Methods:**

Receiver operating characteristic (ROC) analysis was performed for the relationship between TG/HDL-C ratio and accumulation of cardio-metabolic risk factors including visceral obesity, hypertension and diabetes. Logistic regression analysis was performed for the relationships of TG/HDL-C ratio with cardio-metabolic risk factors using the cut-off values obtained by ROC analysis and conventional cut-off values (men, 3.75; women, 3.00).

**Results:**

In ROC analysis, the optimal cut-off values for TG/HDL-C ratio were 2.967 in men and 2.237 in women, which were much smaller than the conventional cut-of values. Odds ratios for multiple cardio-metabolic risk factors of subjects with vs. subjects without a high TG/HDL-C ratio in men and women were 5.75 (4.43–7.46) and 18.76 (10.32–34.13), respectively, by using the new cut-off values and they were 5.03 (3.96–6.39) and 16.11 (9.20–28.20), respectively, by using the conventional cut-off values. The odds ratios for visceral obesity, hypertension and diabetes were comparable when using these two different cut-off values.

**Conclusion:**

Cut-off values should be ideally calculated by ROC analysis. However, the discrimination power of cut-off values for the TG/HDL-C ratio calculated by ROC analysis for cardio-metabolic risk was similar to those by using the conventional cut-off values. Further studies using cardiovascular events as outcomes in the analysis may be needed to determine more suitable cut-off values of the TG/HDL-C ratio.

## Introduction

Early detection of individual cardiovascular risk is important to prevent cardiovascular disease and reduce its mortality. Dyslipidemia, including hyper-LDL cholesterolemia, hypo-HDL cholesterolemia and hyper-triglyceridemia, is a major risk factor of cardiovascular disease. The ratio of LDL cholesterol to HDL cholesterol (LDL-C/HDL-C ratio) is a classical lipid index to predict coronary heart disease [1]. A more recently proposed lipid index for prediction of cardiovascular disease is the ratio of triglycerides to HDL cholesterol (TG/HDL-C ratio), which has been shown to be a better predictor than LDL-C/HDL-C ratio of myocardial infarction [2, 3] and to be associated with insulin resistance [4] and metabolic syndrome [5]. TG/HDL-C ratio has been shown to reflect small dense LDL particles [6], which are more atherogenic than larger buoyant LDL particles [7–10] and has been reported to be an independent risk factor for coronary heart disease [11, 12]. It is a merit that measurement of TG/HDL-C ratio is easy to perform, while measurement of small dense LDL particles requires complicated procedures including ultracentrifugation [13], gradient gel electrophoresis [14] and nuclear magnetic resonance imaging [15]. Thus, TG/HDL-C ratio is a useful index to predict cardiovascular risk in routine and screening examinations.

Both triglycerides and HDL cholesterol are included in the criteria of metabolic syndrome, in which the cut-off value for triglycerides used is 150 mg/dl for men and women and that for HDL cholesterol is 40 mg/dl for men and 50 mg/dl for women [16, 17]. However, various cut-off values for TG/HDL-C ratio have been proposed in previous studies [4, 18–38] as summarized in Table 1. In addition, the cut-off values were different when mg/dl and mmol/L were used as the units of triglycerides and HDL cholesterol: the TG/HDL-C value calculated using mg/dl corresponds to the product of 2.29 and the TG/HDL-C value calculated using mmol/L.

**Table 1:** Previous studies regarding cut-off values of the TG/HDL-C ratio.

Authors, year	Age of subjects (mean or range)	Country	Cut-off values	Units (#1)	Outcome	Ref. no
McLaughlin et al. 2003	50 years (men and women with overweight)	USA	3.0 (men and women)	mg/dl	Insulin resistance	[18]
McLaughlin et al. 2005	Mean years, 42–56 (men and women)	USA	3.5 (men and women)	mg/dl	Insulin resistance	[4]
Cordero et al. 2008	42–44 years (men and women)	Spain	2.75 (men), 1.65 (women)	mg/dl	MS	[19]
Li et al. 2008	20 years or older	USA	3.0 (non-Hispanic whites and Mexican Americans); 2.0 (non-Hispanic blacks) (men and women)	mg/dl	Hyperinsulinemia	[20]
Hadaegh et al. 2010	43.3 years (men); 40.8 years (women)	Iran	4.7 (men), 3.7 (women)	mmol/L	Diabetes	[21]
Kawamoto et al. 2010	60 years (non-obese men and women); 57 (overweight men and women)	Japan	1.50 (non-obese men and women); 2.20 (overweight men and women)	mg/dl	Insulin resistance	[22]
Summer et al. 2010	55 years (African-American men and women)	USA	2.5 (men); indefinable (women)	mg/dl	Insulin resistance	[23]
Arthur et al. 2012	44.23 years (postmenopausal women)	Ghana	0.61 (women)	mmol/L	MS	[24]
Salazar et al. 2012	46 years (men); 45 years (women)	Argentine	3.5 (men); 2.5 (women)	mg/dl	Insulin resistance	[25]
Liang et al. 2013	50 years or older (postmenopausal women)	China	0.88 (women)	mmol/L	MS	[26]
Chen et al. 2014	52.30 years (men, MS-); 51.98 years (men, MS+); 49.14 years (women, MS-); 58.58 years (women, MS+)	China	1.6 (JIS criteria) and 1.2 (ATPIII criteria) (men); 1.1 (both criteria) (women)	mmol/L	MS	[27]
Gasevic et al. 2014	46.8 years (men); 47.5 years (women)	Canada	1.62 (men); 1.18 (women)	mmol/L	MS	[28]
Unger et al. 2014	45 years (MS+); 33 years (MS-)	Argentine	3.1 (men); 2.2 (women)	mg/dl	MS	[29]
Zhang et al. 2015	52.6 years (men, normal weight); 52.2 (men, high weight); 49.8 years (women, normal weight); 54.9 years (women, high weight)	China	1.51 (men); 0.84 (women)	mmol/L	Insulin resistance	[30]
Chen et al. 2016	50.61 years (men, MS+); 48.70 (men, MS-); 53.54 (women MS+); 45.57 (women, MS-)	China	1.10 (men); 0.90 (women)	mmol/L	MS	[31]
Gharipour et al., 2016	50.7 (men and women)	Iran	4.42 (men); 3.76 (women); 3.68 (men and women)	mg/dl	Ischemic heart disease and stroke (#2)	[32]
Li et al. 2016	Mean years: 49.44–53.99 (men); 48.65–56.71 (women)	China	men: 1.3 (HT), 1.3 (DL), 1.4 (DM), 1.4 (RFs); women: 0.9 (HT), 1.0 (DL), 1.0 (DM), 1.1 (RFs)	mmol/L	HT, DL, DM, RFs	[33]
Paulmichl et al. 2016	43.9 years	Austria etc.	2.05 (M value), 1.47 (M/I value)	mg/dl	Insulin resistance	[34]
Song et al. 2016	54.40 years (diabetes); 41.12 years (non-diabetes)	China	1.24 (men and women)	mmol/L	DM	[35]
Abbasian et al. 2017	30–60 years	Iran	4.03 (men); 2.86 (women)	mg/dl	MS	[36]
Kang et al. 2017	9–13 years	Korea	1.41 (men and women)	mg/dl	Insulin resistance	[37]
Deng et al. 2018	68.5 years	China	0.9 (men and women)	mmol/L	Acute ischemic stroke (#2)	[38]

#1, units of triglycerides and HDL cholesterol. The TG/HDL-C value calculated using mg/dl corresponds to the product of 2.29 and the TG/HDL-C value calculated using mmol/L; #2, prospective study. *ATPIII* The Third Adult Treatment Panel, *DL* Dyslipidemia, *DM*, Diabetes mellitus, *HT* Hypertension, *JIS* The Joint Interim Statement, *MS* Metabolic syndrome, *RFs* two or more risk factors, *Ref. no* Reference number

The purpose of this study was to compare two cut-off values of TG/HDL-C ratio as a risk index for cardiovascular disease. One is a cut-off value calculated by using each cut-off value for triglycerides and HDL cholesterol, and the other is a cut-off value obtained by ROC analysis for the relationship between TG/HDL-C ratio and accumulation of three cardio-metabolic risk factors including visceral obesity, hypertension and diabetes. Then the relationships of TG/HDL-C ratio with the risk factors were compared in analyses using the two different cut-off values. There has been, to the best of our knowledge, no report on prediction of incident cardiovascular disease by using the conventional TG/HDL-C ratio calculated as a ratio of the cut-off value for triglycerides to that for HDL cholesterol. Thus, 3.75 (mg/dl/mg/dl) for men and 3.00 (mg/dl/mg/dl) for women, corresponding to 1.64 (mmol/L/mmol/L) for men and 1.31 (mmol/L/mmol/L) for women, are theoretical cut-off values for the TG/HDL-C ratio and are higher than the cut-off values obtained by ROC analysis in most of the previous studies (Table 1) and our present study. However, it was shown in this study that the discrimination power of the cut-off values for high TG/HDL-C ratio calculated by ROC analysis for cardio-metabolic risk is similar to those by using the conventional cut-off values.

## Methods

### Subjects

The subjects were Japanese men (*n* = 6914) and women (*n* = 3282) aged 35–40 years who had received periodic health checkup examinations at workplaces and had been registered in a population-based database in Yamagata Prefecture in Japan. This study was approved by the Ethics Committee of Yamagata University School of Medicine (No. 112 from April 2005 to March 2006, approved on March 13, 2006) and the Hyogo College of Medicine Ethics Committee (No. 3003 in 2018). A population-based database including the results of annual health checkup examinations, in which the participants were not identified, was used, and informed consent from each participant was not obtained in this study. This procedure was approved by the institutional ethics committee. Those who had been receiving drug therapy for dyslipidemia (1.1%) were excluded from subjects of this study. Histories of illness, medication, alcohol consumption, cigarette smoking, and regular exercise (almost every day with exercise for 30 min or longer per day) were surveyed by questionnaires. The subjects were divided into four groups by average cigarette consumption (nonsmokers; light smokers, 20 cigarettes or less per day; heavy smokers, more than 20 and less than 41 cigarettes per day; very heavy smokers, 41 or more cigarettes per day). Average alcohol consumption of each subject per week was reported on questionnaires. The subjects were divided into three groups (nondrinkers, occasional drinkers and regular drinkers) by frequency of drinking. Frequency of weekly alcohol drinking was categorized as “every day” (regular drinkers), “sometimes” (occasional drinkers) and “never” (nondrinkers).

### Measurements

Height and body weight were measured while subjects wore light clothes at the health checkup. Body mass index (BMI) was calculated as weight in kilograms divided by the square of height in meters. Waist circumference was measured at the navel level according to the recommendation of the definition of the Japanese committee for the diagnostic criteria of metabolic syndrome [39]. Waist circumference corrected by height (waist-to-height ratio) has been proposed to be a more reasonable index than waist circumference for abdominal obesity [40]. Waist-to-height ratio has been shown to be a better discriminator than waist circumference or BMI of cardiovascular risk factors and coronary heart disease [41–43]. Therefore, visceral obesity was evaluated by the waist-to-height ratio in this study. The cut-off value of high waist-to-height ratio used was 0.5 [40]. Blood pressure was measured by trained nurses, who were part of the local health-checkup company, with a mercury sphygmomanometer once on the day of the health checkup after each subject had rested quietly in a sitting position. Korotkoff phase V was used to define diastolic pressure. Hypertension was defined as systolic blood pressure of ≥ 140 mmHg and/or diastolic blood pressure of ≥ 90 mmHg. In addition, subjects who were receiving drug therapy for hypertension were included in the hypertensive group regardless of blood pressure levels. Fasted blood was sampled from each subject, and serum triglycerides and HDL cholesterol were measured by enzymatic methods using commercial kits, Pureauto S TG-N and Cholestest N-HDL (Sekisui Medical Co., Ltd., Tokyo, Japan), respectively. TG/HDL-C ratio was calculated as the ratio of triglycerides (mg/dl) to HDL cholesterol (mg/dl), and the conventional cut-off value of high TG/HDL-C ratio was defined as the ratio of the cut-off value of triglycerides (150 mg/dl) to that of HDL cholesterol (40 mg/dl in men and 50 mg/dl in women), namely 3.75 and 3.00 in men and women, respectively.

Blood hemoglobin A1c was used for evaluation of hyperglycemia. Hemoglobin A1c was determined by the latex cohesion method using a commercial kit (Determiner HbA1c, Kyowa Medex, Tokyo, Japan). Hemoglobin A1c values were calibrated by using a formula proposed by the Japan Diabetes Society (JDS) as hemoglobin A1c (National Glycohemoglobin Standardization Program) (%) = 1.02 x hemoglobin A1c (JDS) (%) + 0.25 (%) [44]. Subjects with diabetes were defined as those showing high hemoglobin A1c levels (≥ 6.5%), according to the criteria for diagnosis of diabetes by the American Diabetes Association [45]. Subjects receiving drug therapy for diabetes were also included in the diabetes group. Coefficients of variation for reproducibility of each measurement were ≤ 3% for triglycerides and ≤ 5% for HDL cholesterol and hemoglobin A1c. Subjects with multiple risk factors were defined as those having high waist-to-height ratio, hypertension and diabetes.

### Statistical analysis

Statistical analyses were performed using computer software programs (SPSS version 16.0 J for Windows, Chicago IL, USA and R 2.12.2). Continuous variables were compared between men and women using the unpaired Student’s t test. Since triglycerides and TG/HDL-C ratio did not show normal distributions, they were compared between the groups using the Mann-Whitney U test. Categorical variables were compared using the chi-squared test. How well the TG/HDL-C ratio could discriminate those who did and those who did not have multiple risk factors (visceral obesity, hypertension and diabetes) was evaluated by using a receiver-operating characteristic (ROC) curve. Sensitivity and specificity are the basic measures of accuracy of a diagnostic test: The sensitivity is the probability of a positive test result, while the specificity is the probability of a negative test result. A ROC curve is a plot of sensitivity versus 1 – specificity that offers a summary of sensitivity and specificity across a range of cut-off points for a continuous predictor. The optimal cut-off point was selected by maximizing Youden’s index, which is the difference between the true positive rate (sensitivity) and the false positive rate (1-specificity) in the ROC curve. Discriminability of the TG/HDL-C ratio was measured by the area under the ROC curve (AUC). The 95% confidence interval (CI) for the AUC was estimated by using the DeLong method. To adjust for the optimism of the AUC estimated in our internal cohort, the optimism-corrected AUC and its corresponding 95% CI were also estimated by using a bootstrap method [46]. In logistic regression analysis, odds ratios for visceral obesity, hypertension, diabetes and multiple risk factors were estimated in subjects with vs. subjects without a high TG/HDL-C ratio, defined by using the conventional cut-off values or the cut-off values determined in this study. In multivariate logistic regression analysis, age and histories of smoking, alcohol drinking and regular exercise were adjusted as confounding factors. All probability (*p*) values are 2-sided and values of *p* less than 0.05 were considered to indicate statistical significance.

## Results

### Characteristics of the subjects

Table 2 shows the characteristics of the male and female subjects. Variables related to cardiovascular disease were significantly higher in men than in women except for HDL cholesterol, which was significantly higher in women than in men. There were 310 men (4.48%) and 54 women (1.65%) who showed accumulation of three cardiovascular risk factors (visceral obesity, hypertension and diabetes).

**Table 2:** Characteristics of the subjects

	Overall	Men	Women	*p* value
Number	10196	6914	3282	–
Age (years)	37.5 ± 1.7	37.4 ± 1.7	37.5 ± 1.8	0.458
Smoking (%)	54.1	66.3	28.4	< 0.001
Alcohol drinking (%)	66.7	75.1	49.2	< 0.001
Regular exercise (%)	9.5	11.5	5.2	< 0.001
Therapy for hypertension (%)	1.55	1.88	0.85	< 0.001
Therapy for diabetes (%)	0.69	0.91	0.21	< 0.001
Visceral obesity (%)	32.8	34.3	29.7	< 0.001
Hypertension (%)	14.5	18.4	6.2	< 0.001
Diabetes (%)	1.91	2.39	0.91	< 0.001
Multiple risk factors (%)	3.57	4.48	1.65	< 0.001
Height (cm)	167.4 ± 8.2	171.5 ± 5.9	158.7 ± 5.3	< 0.001
Weight (kg)	65.1 ± 13.6	69.8 ± 12.3	55.0 ± 10.2	< 0.001
Body mass index	23.1 ± 3.9	23.7 ± 3.8	21.8 ± 3.9	< 0.001
Waist circumference (cm)	80.7 ± 10.4	83.0 ± 10.0	75.9 ± 9.7	< 0.001
Waist-to-height ratio	0.483 ± 0.059	0.484 ± 0.057	0.479 ± 0.062	< 0.001
Systolic BP (mmHg)	121.5 ± 15.4	124.6 ± 14.9	114.9 ± 14.4	< 0.001
Diastolic BP (mmHg)	73.0 ± 11.7	75.3 ± 11.5	68.2 ± 10.5	< 0.001
Triglycerides (mg/dl)	91 (60, 148)	111 (73, 175)	63 (47, 91)	< 0.001
HDL cholesterol (mg/dl)	58.8 ± 15.1	55.8 ± 14.7	64.9 ± 14.1	< 0.001
TG/HDL-C ratio	1.58 (0.94, 2.92)	2.02 (1.20, 3.61)	0.98 (0.68, 1.52)	< 0.001
Hemoglobin A1c (%)	5.27 ± 0.59	5.30 ± 0.66	5.21 ± 0.39	< 0.001

Shown are the numbers of subjects, the percentages of subjects, the means with standard deviations for each variable, and the medians with 25th and 75th percentile values indicated in parentheses for each variable. *BP* Blood pressure, *TG/HDL-C ratio* The ratio of triglycerides to HDL cholesterol. Drinkers include both occasional and regular drinkers. *p* values for differences between men and women are also shown

### ROC analysis for the relationships between TG/HDL-C ratio and multiple cardiovascular risk factors

**Fig. 1:**
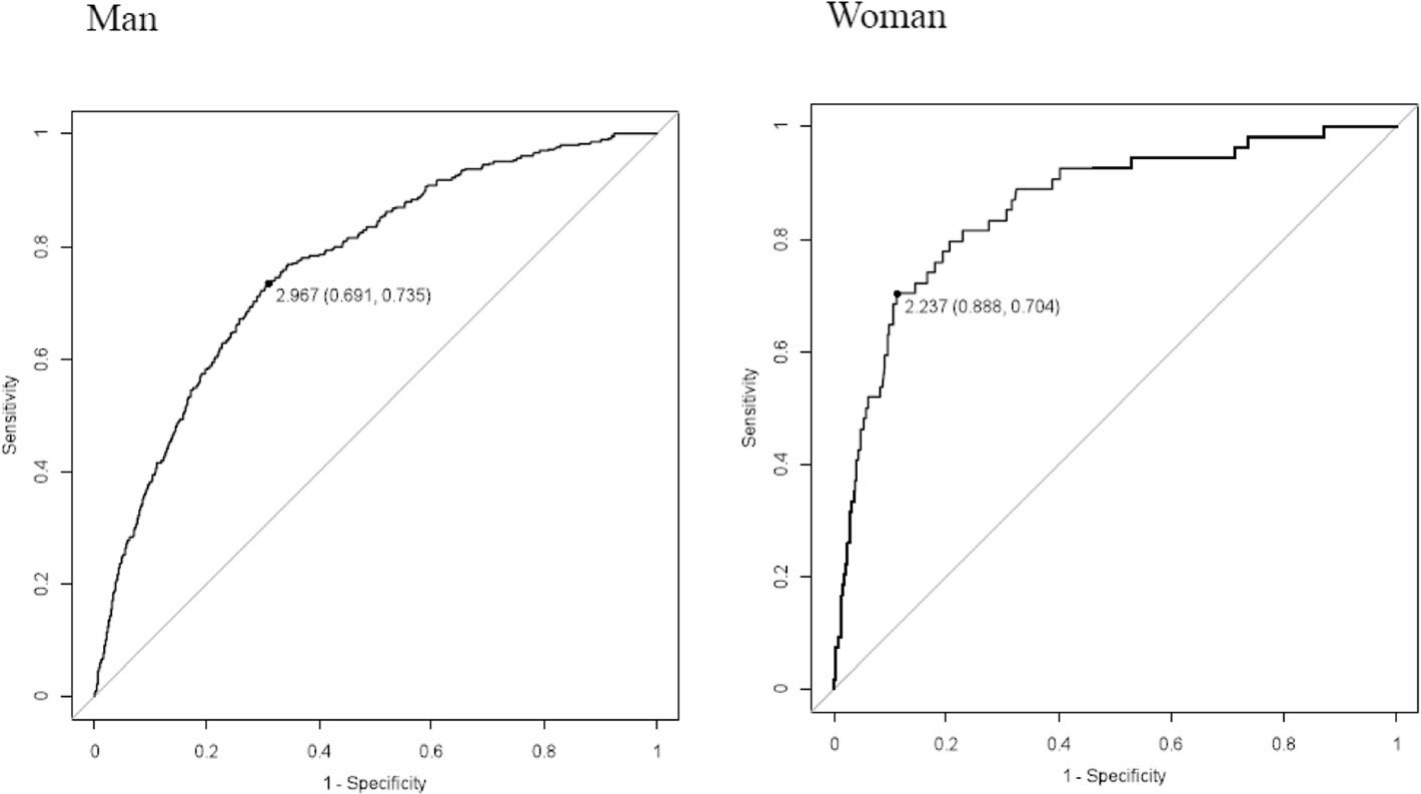
Receiver-operating characteristic (ROC) curves for the relationships between TG/HDL-C ratio and multiple risk factors in men (**A**) and women (**B**). Cut-off values with specificity and sensitivity in parentheses are given in the figures. Area under the ROC curve: men, 0.767 (95% confidence interval: 0.741–0.793); women, 0.858 (95% confidence interval: 0.805–0.910)

Figure 1 shows results of ROC analysis for the relationships between TG/HDL-C ratio and multiple cardiovascular risk factors. The optimal cut-off values of TG/HDL-C ratio for multiple cardiovascular risk factors were 2.967 in men and 2.237 in women. These values are much smaller than the conventional cut-off values (men, 3.75; women, 3.00) calculated by using the known cut-off values of triglycerides and HDL cholesterol. The sensitivity and specificity for each cut-off value are shown in Table 3. The sensitivity and specificity for the conventional cut-off values were lower and higher, respectively, than those for the cut-off values obtained by using ROC analysis.

**Table 3:** Sensitivity and specificity for relationships of multiple risk factors with high TG/HDL-C ratio defined by conventional cut-off values and new cut-off values in men and women.

	Conventional cut-off value Men, 3.75; women 3.00	New cut-off value Men, 2.967; women, 2.237
Men		
Sensitivity	0.606	0.735
Specificity	0.780	0.691
Women		
Sensitivity	0.500	0.704
Specificity	0.943	0.888

We estimated optimism-corrected AUC by using a bootstrap method [46]. Consequently, the optimism-corrected AUC values for the TG/HDL-C ratio in relation to multiple risk factors were 0.764 in men and 0.855 in women and were thus almost the same (but slightly smaller) as the AUC values of 0.767 in men and 0.858 in women shown in Fig. 1, which may be due to a large sample size of the cohort used in this study.

### Logistic regression analysis for the relationships between TG/HDL-C ratio and multiple cardiovascular risk factors

Table 4 shows odds ratios with their 95% confidence intervals of subjects with vs. subjects without a high TG/HDL-C ratio for multiple cardiovascular risk factors in univariate and multivariate logistic regression analyses. In the multivariate analysis, age and histories of smoking, alcohol drinking and regular exercise were used as confounding variables. All of the odds ratios were significantly higher than the reference level of 1.00. Both crude and adjusted odds ratios tended to be higher in the analysis using the new cut-off values of TG/HDL-C ratio obtained by ROC analysis than those in the analysis using the conventional cut-off values of TG/HDL-C ratio.

**Table 4:** Odds ratios for multiple risk factors (visceral obesity, hypertension and diabetes) in subjects with vs. subjects without a high TG/HDL-C ratio defined by conventional cut-off values and new cut-off values in men and women

	Conventional cut-off value Men, 3.75; women 3.00	New cut-off value Men, 2.967; women, 2.237
Men		
Crude	5.45 (4.31–6.89)*	6.23 (4.81–8.06)*
Adjusted	5.03 (3.96–6.39)*	5.75 (4.43–7.46)*
Women		
Crude	16.64 (9.56–28.95)*	18.92 (10.44–34.28)*
Adjusted	16.11 (9.20–28.20)*	18.76 (10.32–34.13)*

Odds ratios with their 95% confidence intervals in parentheses are shown. Asterisks denote significant differences (*p* < 0.01) from the reference level (1.00). In multivariate analyses, age and histories of smoking, alcohol drinking and regular exercise were used as other explanatory variables

### Logistic regression analysis for the relationships of TG/HDL-C ratio with visceral obesity, hypertension and diabetes

Odds ratios of high TG/HDL-C ratio for visceral obesity, hypertension and diabetes are shown in Tables 5, 6 and 7, respectively. In multivariate analysis, age and histories of smoking, alcohol drinking and regular exercise were used as confounding variables (Adjusted). The crude and adjusted odds ratios for visceral obesity, hypertension and diabetes were significantly higher than the reference level in all analyses (Tables 5, 6, 7). Both crude and adjusted odds ratios for visceral obesity, hypertension and diabetes were comparable when using the conventional cut-off values and using the new cut-off values of TG/HDL-C ratio obtained by ROC analysis in this study (Tables 5, 6, 7).

**Table 5:** Odds ratios for high WHtR in subjects with vs. subjects without a high TG/HDL-C ratio defined by conventional cut-off values and new cut-off values in men and women

	Conventional cut-off value Men, 3.75; women 3.00	New cut-off value Men, 2.967; women, 2.237
Men		
Crude	4.06 (3.62–4.56)*	4.17 (3.74–4.64)*
Adjusted	4.02 (3.58–4.52)*	4.13 (3.71–4.61)*
Women		
Crude	6.48 (4.77–8.81)*	5.16 (4.14–6.43)*
Adjusted	6.45 (4.74–8.78)*	5.17 (4.14–6.45)*

Odds ratios with their 95% confidence intervals in parentheses are shown. Asterisks denote significant differences (*p* < 0.01) from the reference level (1.00). In multivariate analyses, age and histories of smoking, alcohol drinking and regular exercise were used as other explanatory variables

**Table 6:** Odds ratios for hypertension in subjects with vs. subjects without a high TG/HDL-C ratio defined by conventional cut-off values and new cut-off values in men and women

	Conventional cut-off value Men, 3.75; women 3.00	New cut-off value Men, 2.967; women, 2.237
Men		
Crude	2.24 (1.97–2.56)*	2.23 (1.97–2.52)*
Adjusted	2.28 (2.00–2.60)*	2.26 (1.99–2.56)*
Women		
Crude	4.72 (3.25–6.84)*	4.23 (3.09–5.79)*
Adjusted	4.75 (3.27–6.90)*	4.29 (3.12–5.88)*

Odds ratios with their 95% confidence intervals in parentheses are shown. Asterisks denote significant differences (*p* < 0.01) from the reference level (1.00). In multivariate analyses, age and histories of smoking, alcohol drinking and regular exercise were used as other explanatory variables (Adjusted)

**Table 7:** Odds ratios for diabetes in subjects with vs. subjects without a high TG/HDL-C ratio defined by conventional cut-off values and new cut-off values in men and women

	Conventional cut-off value Men, 3.75; women 3.00	New cut-off value Men, 2.967; women, 2.237
Men		
Crude	4.44 (3.25–6.08)*	4.38 (3.15–6.09)*
Adjusted	4.06 (2.95–5.57)*	3.99 (2.86–5.56)*
Women		
Crude	11.86 (5.68–24.77)*	11.34 (5.42–23.72)*
Adjusted	11.73 (5.55–24.77)*	11.38 (5.40–24.00)*

Odds ratios with their 95% confidence intervals in parentheses are shown. Asterisks denote significant differences (*p* < 0.01) from the reference level (1.00). In multivariate analyses, age and histories of smoking, alcohol drinking and regular exercise were used as other explanatory variables (Adjusted)

## Discussion

Dyslipidemia is known to be closely associated with other major atherosclerotic risk factors such as obesity, hypertension and diabetes [47]. In order to determine the cut-off values for the TG/HDL-C ratio, a known cardiovascular risk index, ROC analysis was performed using accumulation of multiple risk factors, including visceral obesity, hypertension and diabetes, as a dependent variable in early middle-aged men and women. The optimal cut-off values obtained by the analysis were 2.967 in men and 2.237 in women, which are much smaller than the conventional cut-off values, 3.75 in men and 3.00 in women, simply calculated by using each of cut-off values for triglycerides (150 mg/dl in men and women) and HDL cholesterol (40 mg/dl in men and 50 mg/dl in women). In this study, we for the first time investigated whether the discriminating power of the TG/HDL-C ratio is different in analyses using the above two different cut-off values. The odds ratios for multiple risk factors of subjects with vs. subjects without a high TG/HDL-C ratio were slightly higher in the analysis using the cut-off values determined in this study than in the analysis using the conventional cut-off values (Table 4). When each component of the multiple risk factors, including visceral obesity, hypertension and diabetes, was used as an outcome variable in ROC analysis, the odds ratios of subjects with vs. subjects without a high TG/HDL-C ratio in men or women were not different in the analyses using the new and conventional cut-off values. Thus, similar discriminating power of the TG/HDL-C ratio for each of the components of visceral obesity, hypertension and diabetes was obtained by these different cut-off values, although the values were considerably different.

There is a gender difference in HDL cholesterol level, which is higher in women than in men, and different cut-off values for men and women are in fact used in the NCEP criteria for metabolic syndrome [17]. Therefore, it is reasonable that there is also a gender difference in cut-off values of the TG/HDL-C ratio: The values were larger in men than in women (Table 1). Thus, it is preferable to use different cut-off values of the TG/HDL-C ratio for men and women. The cut-off values for men and women estimated in the present study are relatively close to those reported by an Argentine group but are quite different from the values reported by a Japanese group (Table 1). There have been seven reports from China in which there were various cut-off values of the TG/HDL-C ratio (Table 1). Thus, there seems to be no ethnicity-related difference in the cut-offs of TG/HDL-C ratio. Age is also an important factor to determine the cut-off values, and mean ages of participants were different among the previous studies. The outcome in ROC analysis is also a determinant of the cut-off values, and insulin resistance or metabolic syndrome was an outcome used in ROC analysis in most of the previous studies (Table 1). Thus, differences in age and gender of subjects and outcome in ROC analysis might have caused the differences in cut-off values for the TG/HDL-C ratio in previous studies.

Regarding the utility of the TG/HDL-C ratio in individual risk of cardiovascular disease, dyslipidemia is a potential (usually asymptomatic) risk factor of cardiovascular disease and is often found by blood examinations. Dyslipidemia is associated with other overt cardiovascular risk factors such as obesity, hypertension and diabetes. Not only elderly people but also younger adults are affected by dyslipidemia: About 31% of adults aged 20 to 29 years and 38% of adults aged 30 to 39 years in US showed HDL cholesterol and/or triglyceride levels that were out of the ranges of levels recommended by NCEP [48]. Thus, evaluation of dyslipidemia using the TG/HDL-C ratio in an early stage of individual life and earlier correction of lifestyle are useful for predicting the occurrence of obesity, hypertension and diabetes and for preventing future cardiovascular events. However, the appropriate cut-off value for the TG/HDL-C ratio has remained to be determined.

A cut-off value should be ideally determined by ROC analysis. The cut-off value for the TG/HDL-C ratio depends on its associated outcome, and an outcome yielding a larger AUC in ROC analysis is preferable to determine the cut-off value of the TG/HDL-C ratio. A single TG/HDL-C ratio can be created by varying different combinations of triglyceride and HDL cholesterol values. The TG/HDL-C ratio level that optimally discriminated the risk of cardiovascular disease could be identified from these varying combinations. Thus, the predicted cardiovascular risks for the groups classified by using the conventional and the newer cut-off values of TG/HDL-C ratio could be contrasted. However, these cut-off values were not greatly different in their discriminating power for cardiovascular risk in the present study.

In the present study, AUC values for the TG/HDL-C ratio in relation to multiple risk factors were 0.767 in men and 0.858 in women (Fig. 1), which are generally evaluated as moderate accuracy (AUC: 0.7 ~ 0.9). In most previous studies, cut-off values of the TG/HDL-C ratio were obtained by ROC analysis using variables such as metabolic syndrome and insulin resistance that were associated cross-sectionally with the TG/HDL-C ratio [4, 18–31, 33–37], and there have been only a few prospective studies on cut-off values of TG/HDL-C ratio. In recent reports from Iran and China [32, 38] using prospective cohorts with outcomes of ischemic heart disease and ischemic stroke, 3.68 mg/dl/mg/dl and 2.06 mg/dl/mg/dl (corresponding to 0.9 mmol/L/mmol/L) were proposed as cut-off values of the TG/HDL-C ratio in men and women, although the AUC in those studies were not large (0.575 and 0.647). In the present study, cut-off values of the TG/HDL-C ratio, obtained by ROC analysis using accumulation of multiple risk factors as an independent outcome variable, did not show strong discriminating power for cardiovascular risk since similar odds ratios for cardiovascular risk factors were obtained in logistic regression analysis using the new cut-off values and conventional cut-off values in men and women. Therefore, the cut-off value of the TG/HDL-C ratio, as a cardiovascular risk index representing dyslipidemia, may need to be determined in prospective studies by using a more suitable outcome such as cardiovascular events in ROC analysis with high accuracy.

There are limitations of this study. The subjects were relatively young, and further studies are needed to test the influence of age on the cut-off value of TG/HDL-C ratio in ROC analysis. To our knowledge, there has been only one study on cut-off values for Japanese, in which the optimal cut-off values were calculated to be 1.50 in non-obese men and women (mean age of 60 years) and 2.20 in overweight men and women (mean age of 57 years) [22]. These cut-off values are much lower than that in the present study, and one possible reason for this is a difference in ages of subjects. Age and lifestyles including habits of smoking, alcohol drinking and regular exercise were adjusted in multivariate logistic regression analysis in the present study. However, there are other possible confounding factors, e.g., diet, nutrition and socio-economic status, influencing the relationships between TG/HDL-C ratio and cardiovascular risk factors. All of the subjects in this study were Japanese. A racial difference in the TG/HDL-C ratio has been suggested [20]. Another limitation is that only a single reading of blood pressure on a single day was used as blood pressure measurement to determine hypertension status. This study was cross-sectional in its design, and further prospective studies are needed to discuss causal relationships between the TG/HDL-C ratio and the risk of cardiovascular disease. Since the newer cut-off values are lower than the conventional cut-off values, the sensitivity and specificity of the newer cut-off values are, due to a trade-off, higher and lower, respectively, than those of the conventional cut-off values. Finally, as mentioned above, ROC analysis using a more suitable outcome is needed to determine the cut-off value of the TG/HDL-C ratio that discriminates cardiovascular risk more effectively.

## Conclusions

We investigated two cut-off values of the TG/HDL-C ratio, a conventional cut-off value and a cut-off value obtained by ROC analysis. The power of these cut-off values to discriminate each of the cardio-metabolic risk factors, including visceral obesity, hypertension and diabetes, was similar, and further prospective studies using cardiovascular events as independent variables in ROC analysis may be needed to determine more suitable cut-off values of TG/HDL-C ratio.

## Data Availability

The datasets used and/or analyzed during the present study are available from the corresponding author on reasonable request.

## Abbreviations

ANCOVA: Analysis of covariance
AUC: Area under the ROC curve
BMI: Body mass index
CI: Confidence interval
HDL-C: High density lipoprotein cholesterol
JDS: The Japan Diabetes Society
LDL-C: Low density lipoprotein cholesterol
LDL-C/HDL-C: The ratio of LDL cholesterol to HDL cholesterol
RF: Risk factors
ROC: Receiver operating characteristic
TG: Triglycerides
TG/HDL-C: The ratio of triglycerides to HDL cholesterol
